# Genome-Wide Identification of the *CtNF-Y* Gene Family and Expression Analysis of Different Flower Colours and Different Flowering Stages in *Carthamus tinctorius* L.

**DOI:** 10.3390/plants14142111

**Published:** 2025-07-09

**Authors:** Jianhang Zhang, Shuwei Qin, Lili Wang, Mengyuan Ma, Wanting Yang, Wenjie Shen, Yaqian Lu, Mingqiang Bao, Meng Zhao, Hongbin Li, Asigul Ismayil, Aiping Cao

**Affiliations:** Key Laboratory of Xinjiang Phytomedicine Resource and Utilization of Ministry of Education, Xinjiang Production and Construction Corps Key Laboratory of Oasis Town and Mountain-Basin System Ecology, College of Life Sciences, Shihezi University, Shihezi 832003, China; zjh17630412656@163.com (J.Z.); qsw2623201516@163.com (S.Q.); lfyroquz@163.com (L.W.); mengyuanma2022@163.com (M.M.); 15138409277@163.com (W.Y.); 18919309525@163.com (W.S.); 18894489795@163.com (Y.L.); 20242006076@stu.shzu.edu.cn (M.B.); zm022362@163.com (M.Z.)

**Keywords:** *NF-Y*, safflower, flower colour, flowering time, nucleus

## Abstract

Safflower (*Carthamus tinctorius* L.) is a plant in the family of Asteraceae, and the dried tubular flowers are used as medicine, which contain active ingredients such as safflower yellow pigment and safflower glycosides. They play important roles in many fields. NF-Y, as an important transcription factor in plants, regulates a variety of plant life activities. In this study, we identified and analysed 11 *CtNF-Y* gene family members from safflower for the first time. Their core motifs, which are conserved structural domains, gene structures, and cis-acting elements, are described in this study. In addition, there was good collinearity between safflower *CtNF-Y* and other species. Protein–protein interaction network analysis showed that the CtNF-YA1 and CtNF-YB subfamilies were the core proteins of the interaction network. Real-time quantitative PCR (qRT-PCR) studies showed that the expression level of the *CtNF-Y* gene was regulated by safflower flower colour and safflower flowering period. Subcellular localisation results showed that three CtNF-Y proteins were located in the nucleus, the cellular regulatory centre of the plant. This study will provide valuable insights into the selection of key candidate genes in the network of regulatory mechanisms for the formation of safflower flower colour and flowering time.

## 1. Introduction

Safflower (*Carthamus tinctorius* L.) is a versatile cash crop whose flowers and seeds are widely used in traditional herbal medicine in China, Korea, Japan, and other Asian countries [1]. At least 104 compounds, mostly flavonoids, alkaloids, and alkynes, have been isolated and identified from the plant. Modern pharmacological experiments have proven that safflower and its active ingredients have a wide range of biological activities, including anticoagulant, antioxidant, anti-inflammatory, antihepatic fibrotic, and anti-tumour effects. They are commonly used in the treatment of anaemia, diabetes, and stroke [2]. With the deepening of research, there are more and more relevant reports on the active ingredients of safflower. The primary extract of safflower significantly attenuated ischemic damage to brain tissue and improved neurological function scores. In addition, safflower reduced the degree of apoptosis in brain tissue, regulated the expression of apoptosis-related factors (Bcl-2, Bax, and Caspase 3), and markedly decreased the expression of MMP-9 while increasing the expression of tissue inhibitor of metalloproteinases 1 (TIMP1) protein, suggesting that the safflower has a protective effect on brain injury [3]. Moreover, it has been shown that safflower can exert anti-hepatic fibrosis and inhibit the activation of hepatic stellate cells (HSCs) by inhibiting the PI3K/Akt/mTOR pathway [4]. As a pharmacological plant, safflower has a decisive position in the field of pharmacology in China and even in the world. However, there is still a lot of genetic information about safflower, which greatly increases the difficulty of research on safflower genes and its active ingredients. In recent years, with the development of molecular biology technology, the analysis of safflower genetic information has become a hot topic, which is important for the prediction of safflower genes. However, up to now, the biological information of safflower is still rare.

The discovery of the nuclear factor Y (NF-Y) originated from the study of the CCAAT-box, also known as CCAAT box-binding factor (CBF) or haemoglobin-activating protein (HAP), which is a highly conserved trimeric transcription complex found in all eukaryotic organisms [5,6]. Early researchers found that the NF-Y transcription factors of most organisms are divided into three main subfamilies: NF-YA (HPA2), NF-YB (HPA3), and NF-YC (HAP5), but a fourth subfamily, HAP4, has been identified in fungi such as yeast, which is regulated by carbon sources and has an important role in respiratory metabolism in fungi [7,8]. In recent years, significant progress has also been made in elucidating the biological functions of plant NF-Y. The earliest study of NF-Y in plants came from *Arabidopsis thaliana* on LEC1 (At NF-YB8), a key factor in embryonic development. It is involved in different biological processes, such as embryogenesis, morphogenesis, seed dormancy, and dehydration tolerance [9,10,11,12]. Some studies have reported that OsNF-YB7 and OsNF-YB9 in rice play equally important roles in the regulation of seed development, and OsNF-YB9 can interact with the bZIP family transcription factor OsbZIP76 to affect the process of endosperm cellularisation in *Oryza sativa*. In addition, OsNF-YB9 mutations resulted in altered seed kernel type, increased chalkiness, and disrupted expression of starch synthesis-related genes in the mutants. Meanwhile, OsNF-YB9 interacted with SPK, a calcium-dependent protein kinase that is specifically expressed in the endosperm. Unlike OsNF-YB9, which is only expressed in the rice endosperm, OsNF-YB7 is specifically expressed in the embryo at early stages of development, and knockdown or disruption of OsNF-YB7 can lead to embryonic lethality [13,14,15]. In addition to regulating seed development, NF-Y transcription factors also play a very important role in regulating photoperiodic flowering, and the overexpression of *PtNF-YA9* in *Populus* resulted in slow stem elongation and delayed flowering [16]. ZmNF-YA3 in maize (*Zea mays* L.) promotes flowering by binding to the ZmFT-like12 promoter region [17]. AtHAP3b is an important flowering regulator and can affect flowering time by regulating the expression of FLOWERING LOCUS T (FT) in *A. thaliana* [18]. NF-Y also promotes the *SUPPRESSOR OF OVEREXPRESSION OF CONSTANS* (SOC1)by binding to Teosinte Branched 1/Cycloidea/Proliferating Cell Factor (TCP7) and REPRESSOR OF ga1-3 (RGA) promoter transcriptionally regulates flowering in *A. thaliana* [19]. NF-Y is equally vital for plants to cope with biotic and abiotic stresses. AtABRE binding factors ABF3 and ABF4 contribute to the completion of the life cycle of *Arabidopsis* under drought conditions by interacting with NF-YC3/4/9 to promote flowering by inducing SOC1 transcription under drought conditions [20]. Overexpression of *NF-YB2* and *NF-YB3* in *Arabidopsis* specifically enhances plant tolerance to drought and heat stress, but nf-yb2 and nf-yb3 mutants are less tolerant to drought and heat stress [21]. *AtHAP5A* regulates freezing stress resistance in *Arabidopsis thaliana* by binding to the CCAAT motif of *AtXTH21*. Plants overexpressing *AtHAP5A* were more tolerant to freezing stress than wild-type plants, whereas AtHAP5A loss-of-function mutants were more sensitive to freezing stress than wild-type plants. Similarly, plants overexpressing *AtXTH21* showed better freezing resistance, whereas the *xth21* knockout mutant showed poorer freezing resistance [22]. Not only that, NF-Y also has a positive effect on the osmotic pressure alteration in response to salt stress in Arabidopsis thaliana [23,24]. It has been found that NF-YB2 and NF-YB3 enhance plant resistance to the necrotrophic pathogen *Botrytis cinerea* by positively regulating the transcription of JA-responsive genes and by negatively regulating the abundance of JASMONATE-ZIM DOMAIN (JAZ) protein [25].

Although most research on NF-Y has focused on *Arabidopsis*, recent studies have shown that NF-Y plays a similar role in other plant species. The *SlNF-YA3b* gene regulates tomato flowering time, and knockdown of *NF-YA3b* results in an early flowering phenotype, whereas the overexpression of *NF-YA3b* delays flowering time in transgenic tomato plants, which can be applied to crop flowering management [26]. PhNF-YC2 and PhNF-YC4 in *Petunia hybrida* regulate flowering time [27]. The *GmNF-YA5* gene enhances drought tolerance in soybean (*Glycine max* L.) [28]. Homoplastically, the *ZmNF-YC12* gene regulates drought tolerance and resilience in maize, whereas silencing *ZmNF-YC12* weakens drought tolerance and resilience in maize [29]. It has been found that the *NtNF-YC4* gene of *Nicotiana tabacum* regulates plant starch biosynthesis in response to insect pests, which in turn leads to increased resistance of tobacco to common pests such as *Bemisia tabaci* and *Myzus persicae* [30,31]. In recent years, although there have been many reports on NF-Y transcription factors in many fields and plants, little is known about the *NF-Y* genes in safflower, and it is extremely important to analyse the functions of the *NF-Y* gene family in safflower.

Studies have shown that NF-Y is an important transcription factor in plants, which is involved in salt tolerance, drought resistance, cold resistance, and disease resistance. However, there is no report on the study of NF-Y transcription factors in safflower. In this study, we identified 11 CtNF-Y family members in safflower using bioinformatics, and analysed their physicochemical properties, chromosomal localisation, gene structure, covariance analysis, and protein interactions network. This will provide a theoretical basis for future in-depth studies on the function and regulatory mechanism of *CtNF-Y* genes.

## 2. Results

### 2.1. Identification of the CtNF-Y Gene Family in Carthamus tinctorius *L.*

Based on the genomic data of safflower [32], the published NF-Y protein sequences in *Arabidopsis thaliana* were used as reference sequences and searched by HMM. A total of 11 members of the *CtNF-Y* gene family were identified after removal of duplicates and structural domain identification. In order to distinguish these genes, the genes were categorised and named according to their subclade and chromosomal location: CtNF-YA1~CtNF-YA3, CtNF-YB1~CtNF-YB5, and CtNF-YC1~CtNF-YC3, as shown in Supplementary Table S1. By analyzing physicochemical properties, the amino acid lengths of the 11 CtNF-Ys ranged from 130 aa (CtNF-YB2) to 354 aa (CtNF-YA3). CtNF-YA is the longest, ranging from 213 aa (CtNF-YA1) to 354 aa (CtNF-YA3), with an average length of 287 aa, followed by CtNF-YC, which ranged from 261aa (CtNF-YC1) to 267 aa (CtNF-YC2), with an average length of 263.3 aa, and CtNF-YB, which is the shortest, ranging from 130 aa (CtNF-YB2) to 180 aa (CtNF-YB1), with an average length of 159.6 aa, and the amino acid lengths have obvious differences. The molecular weights ranged from 14.59 kDa (CtNF-YB2) to 38.93 kDa (CtNF-YA3), their predicted isoelectric points ranged from 5.05 (CtNF-YC3) to 8.63 (CtNF-YA3), and the instability index ranged from 33.85 (CtNF-YB2) to 75.90 (CtNF-YC3). All CtNF-Y proteins were expected to be localised in the nucleus, and most family members had an instability index of more than 40, indicating that they are unstable. The Aliphatic index ranged from 52.24 (CtNF-YB4) to 68.73 (CtNF-YC2), and the GRAVY ranged from −1.050 (CtNF-YA1) to −0.583 (CtNF-YC1). All CtNF-Y members have more hydrophilic regions than hydrophobic regions (Supplementary Figure S1), suggesting that they may be hydrophobic proteins, indicating that the CtNF-Y family members are all hydrophilic proteins. In addition, analysis of the transmembrane structural domains of the CtNF-Y amino acid sequences (Supplementary Figure S2) showed that all amino acids of these 11 CtNF-Y members are outside the membrane, suggesting that the synthesis of these proteins can function directly without transmembrane transport. Prediction and analysis of the signal peptides of the CtNF-Y protein members (Supplementary Figure S3) showed that none of them had signal peptides and were non-secretory proteins.

### 2.2. Secondary and Three-Dimensional Structures Analysis of the CtNF-Ys

The secondary structure of 11 CtNF-Y proteins was predicted and analyzed, and the results are shown in Supplementary Table S2. α-helixs and random coils are the main structures, but the extension chain and β-turn are scattered throughout the protein. Among them, the α-helix occupies 12.59% (CtNF-YA2)-56.15% (CtNF-YB2), the β-turn occupies 1.02% (CtNF-YA2)-4.70% (CtNF-YB5), the extended strand occupies 0.77% (CtNF-YB2)-5.62% (CtNF-YC2), and the random coil chain accounts for 39.23% (CtNF-YB2)-83.33% (CtNF-YA2). Then, protein three-dimensional structure prediction was performed for all CtNF-Y family members (Supplementary Figure S4) and matched the secondary structure prediction.

### 2.3. Conserved Motif, Domain, and Gene Structure of the CtNF-Y in Safflower

**Figure 1:**
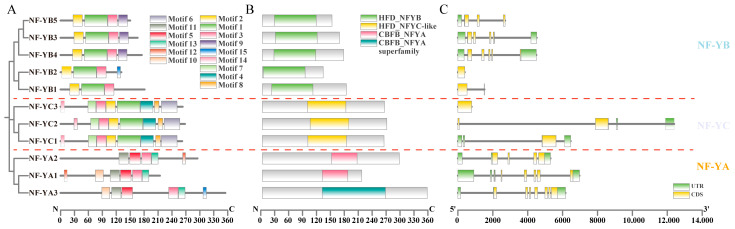
Phylogenetic relationship, motif composition, conserved domains and gene structure of the CtNF-Y family proteins. (**A**) The phylogenetic tree and the motif composition in CtNF-Ys. Different-coloured boxes represent putative motifs; the lengths of motifs in each protein are presented proportionally. The phylogenetic tree was constructed using the Neighbour-Joining (NJ) method. (**B**) The HFD_NFYB, HFD_NFYC-like and CBFB_NFYA superfamily domain of CtNF-Ys. (**C**) The exon-intron structure of the CtNF-Y genes. Green boxes indicate 5′ UTR and 3′ UTR, yellow boxes indicate exons, and black lines indicate introns. N—N-terminus of a protein; C—C-terminus of a protein.

The MEME and Pfam websites were employed to identify the conserved motifs and domains of these CtNF-Ys (Figure 1). The CtNF-Y family was divided into three subfamilies, A, B, and C. Conserved motif analysis of CtNF-Y proteins showed that the CtNF-YA family contained 7 conserved motifs, the CtNF-YB family contained 5 conserved motifs, and the CtNF-YC family contained 9 conserved motifs (Figure 1A). Among them, motif 3, motif 5, motif 11, and motif 13 are present in all NF-YA proteins; subfamily B contains motif 9 except CtNF-YB1 and CtNF-YB2; and subfamily C contains seven identical motifs, but motif 3 is only found in CtNF-YC2, which is presumed to be related to the specific binding of DNA. All CtNF-YA proteins contained the core conserved structural domain of CBFB_NF-YA (Figure 1B), all CtNF-YB proteins contained the HFD_NFYB structural domain, and all CtNF-YC proteins contained the HFD_NFYC structural domain.

To gain further insights into the structural diversity of *CtNF-Ys*, the exon/intron organisations of *CtNF-Y* genes were determined by the TBtools v1.099 software (Figure 1C). Members in the same subfamily are more similar in structure, among which *CtNF-YAs* all contain 4-7 exons, *CtNF-YB2* and *CtNF-YC3* have no introns, among which the gene structures of *CtNF-YB3* and *CtNF-YB4*, as well as all *CtNF-YC* family members are almost identical, suggesting that the gene structures of the members of the *CtNF-Y* family are consistent with the evolutionary relationships remain consistent.

### 2.4. Cis-Acting Elements Analysis of the CtNF-Y Gene Family

**Figure 2:**
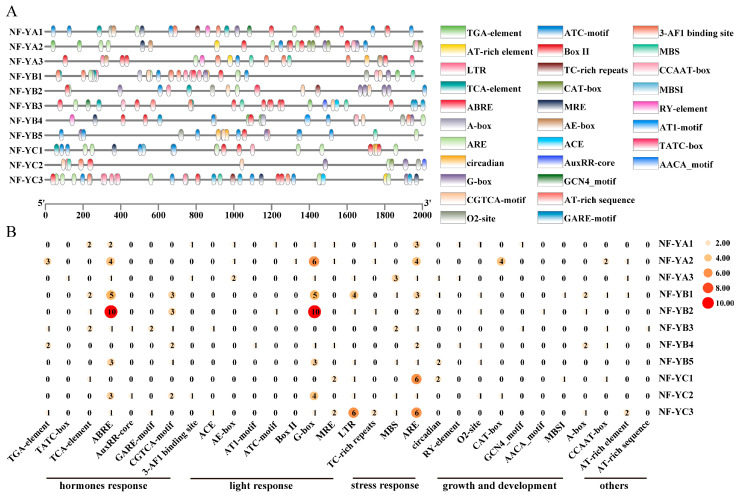
Predicted cis-elements of the promoters of *CtNF-Ys*. (**A**) Binding sites of all cis-elements on promoters of *CtNF-Ys*. (**B**) Number and classification heatmap of *CtNF-Ys* cis-elements.

Cis-acting elements in the promoter region of a gene influence the pattern of gene expression. Therefore, to gain a preliminary understanding of the roles of the 11 *CtNF-Y* genes, we identified a total of 30 potential cis-elements in the promoter region (Figure 2).

The analysis of CtNF-Y promoter cis-acting elements in this study showed that the *CtNF-Y* gene promoter acting elements can be divided into five major categories: hormone-related, including growth hormone-responsive element (AuxRR-core and TGA-element), MeJA-responsive element (CGTCA-motif), salicylic acid-responsive element (TCA-element) and gibberellin-responsive element (GARE-motif, P-box, and TATC-box), respectively, and gibberellin-responsive elements (GARE-motif, P-box, and TATC-box). Light-responsive ones include eight response elements such as the G-box, ACE, AT1-motif, and ATC-motif. Stress-related, including anaerobic induction element (ARE), defense and stress responsiveness element (TC-rich repeats), low-temperature responsiveness element (LTR), and drought-inducibility element (MBS). Growth and development-related, including seven elements, such as the CAT-box involved in meri-stem expression, the O2-site involved in zein metabolism regulation, and the RY-element involved in seed-specific regulation. In addition, the GCN4-motif is a cis-element that is highly conserved in cereal crop seed storage protein promoters. There are some other elements, such as the A-box, CCAAT-box, and AT-rich element (Figure 2A). In the analysis of abiotic stress-related cis-acting elements, it was found that all *CtNF-Y* gene family members contained the ARE antioxidant cis-acting element (Figure 2B); among the hormone-corresponding elements, 10 *CtNF-Y* members contained one or more abscisic acid-responsive cis-acting elements, ABRE, and, more strikingly, all members of the *CtNF-YA* subfamily contained the ABRE cis-acting element, which was higher than that of the *CtNF-YB* subfamily (4/5) and *CtNF-YC* subfamily (2/3), suggesting that *CtNF-Y* may be extensively involved in the drought-responsive pathway. Interestingly, among the light-responsive elements, the G-box, which usually acts synergistically with other cis-acting elements to regulate gene-induced expression, has a similar situation. During growth and development, only two subfamilies, *CtNF-Y* (*CtNF-YA1* and *CtNF-YA3*) and *CtNF-YB* (*CtNF-YB4*), contain the RY-element, and some members of all three *CtNF-Y* subfamilies contain O_2_-sites, suggesting that different *CtNF-Ys* have different roles in plant growth.

### 2.5. Phylogenetic Analysis of CtNF-Y Proteins

**Figure 3:**
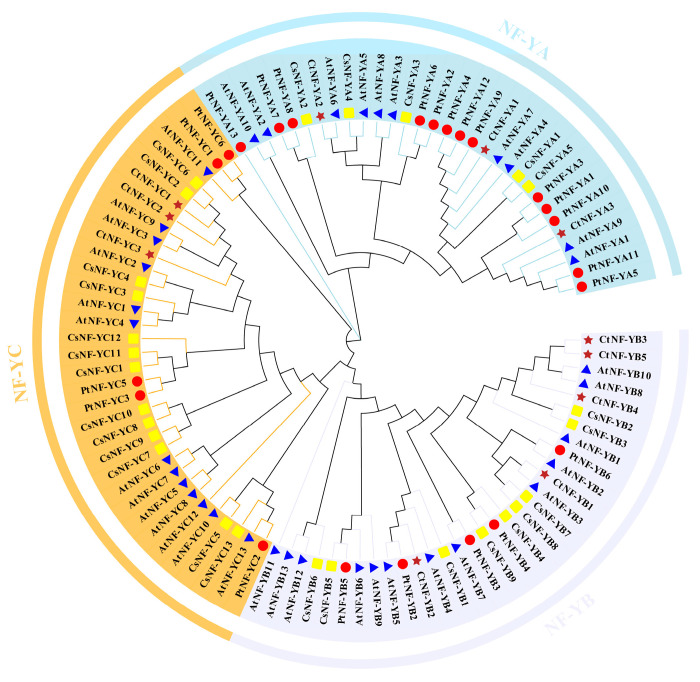
Phylogenetic analysis of CtNF-Y proteins in *C. tinctorius* L. Phylogenetic analyses were conducted on CtNF-Y proteins from safflower (brown stars), *Arabidopsis* (blue triangles), poplar (red circles), and orchid (yellow squares). ClustalW was used for multiple sequence alignment. Phylogenetic trees were constructed using the Neighbour-Joining (NJ) method with 1000 bootstrap repeats.

To investigate the phylogenetic relationships among NF-Y proteins from representative monocotyledonous and dicotyledonous plants, we constructed phylogenetic trees for four species, including 11 CtNF-Y proteins, 36 AtNF-Y proteins, 23 PtNF-Y, and 26 CsNF-Y proteins. All species NF-Y proteins were named based on published data. The neighbour-joining (NJ) method was used to build a phylogenetic tree (Figure 3). Phylogenetic analysis showed that the 96 NF-Y members could be categorised into three clades, NF-YA, NF-YB, and NF-YC. The analysis revealed that all members of the CtNF-YA, PtNF-Y, CsNF-Y, and AtNF-YA proteins are located in the same clade, and CtNF-YB and CtNF-YC are also similarly classified with the other species, respectively, a lineage-evolutionary relationship that suggests that they may be relatively conserved and have similar biological functions.

### 2.6. Chromosomal Distribution and Collinearity Analysis of the CtNF-Y Gene Family

**Figure 4:**
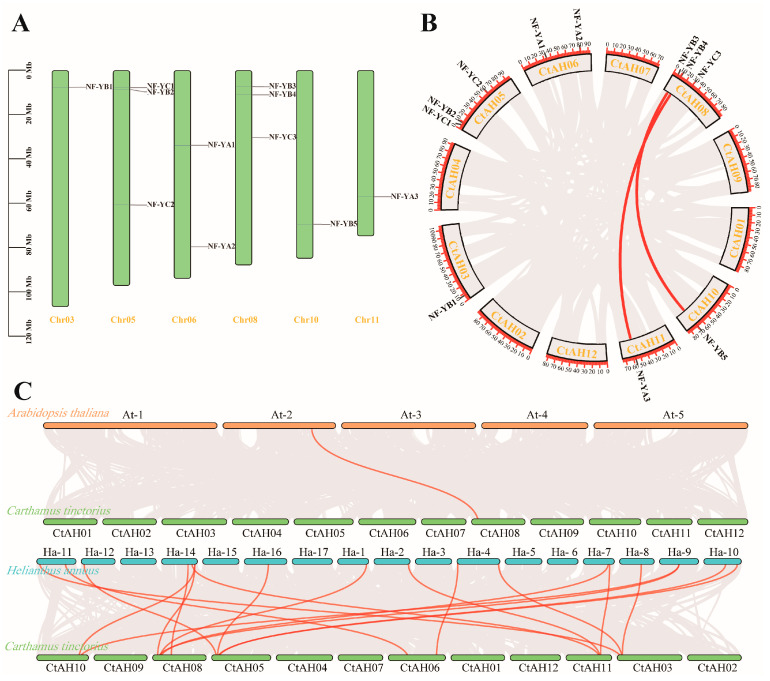
Chromosome and collinearity analysis of *CtNF-Y* genes. (**A**) Chromosome distribution of *CtNF-Y* genes. (**B**) Intraspecific synteny analysis of *CtNF-Y* genes in safflower. (**C**) Interspecific collinearity analysis of *CtNF-Y* genes. The red lines indicate segmentally duplicated gene pairs and orthologous gene pairs. The chromosome number is indicated at the top of each chromosome.

To observe the distribution of the *CtNF-Y* gene family members in chromosomes, we investigated chromosomal localisation and the results are shown in Figure 4A. Chromosomal localisation showed that the 11 *CtNF-Y* genes were distributed on 6 chromosomes. Among them, *CtNF-YA* gene members were located on chromosomes 6 and 11, respectively; *CtNF-YB* gene members were located on chromosomes 3, 5, 8, and 10, respectively; and *CtNF-YC* gene members were located on chromosomes 5 and 8, respectively. The highest number of genes was distributed on chromosomes 5 and 8.

Collinearity analysis can be a good explanation for homology between genes, and collinear homologous sequences may have similar functions, so we performed collinearity analysis of the *CtNF-Y* genes in the safflower genome. Intragenomic homology analysis showed that duplication relationships exist in the safflower genome (Figure 4B). Among the 11 *CtNF-Y* genes, two segmental duplication events were identified, and intraspecific collinearity existed between *CtNF-YA3* and *CtNF-YB4*, and *CtNF-YB5* and *CtNF-YB3*, respectively, and these duplication events were distributed on chromosomes 8, 10, and 11 (Figure 4A). It was hypothesised that these genes may have similar functions. Homology analysis of safflower and *Arabidopsis* revealed one homologous gene pair. In addition, there were 16 collinear gene pairs between safflower and sunflower (Figure 4C), suggesting that the *CtNF-Y* genes are conserved across species.

### 2.7. Protein Interaction Network Analysis of the CtNF-Y Family

**Figure 5:**
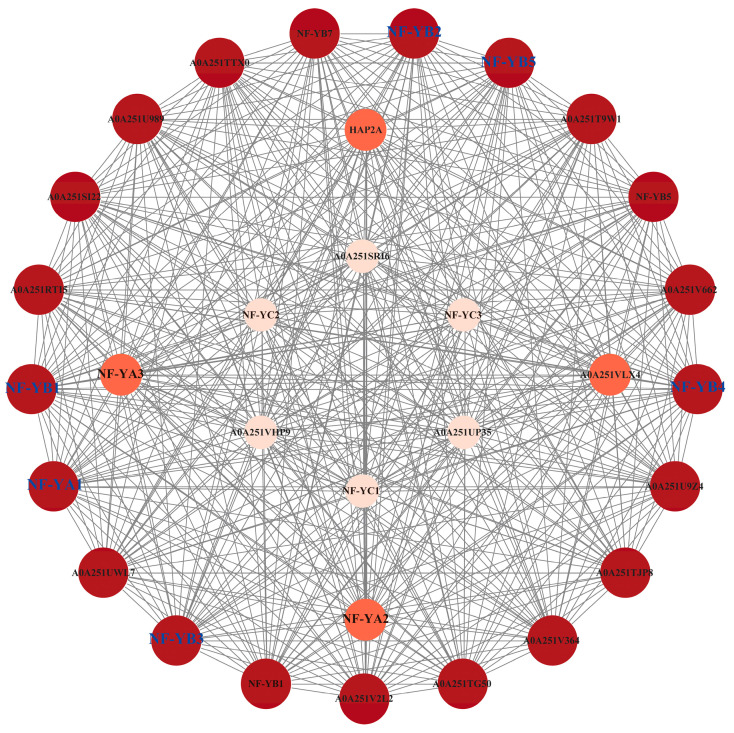
Protein interaction network of safflower NF-Y family members. The black line indicates an interaction between the two.

To analyse potential interactions of CtNF-Y proteins with other proteins, we predicted proteins interacting with the safflower CtNF-Y family using the STRING website to identify the highest-scoring homologues. The results were visualised using Cytoscape (version 3.10.0) (Figure 5). The entire protein interaction network has 457 edges and 31 nodes, and each protein node has certain interactions with other protein nodes. There are 20 proteins located outside the interaction network, among which the CtNF-Y members are the CtNF-YA1 and CtNF-YB subfamilies (CtNF-YB1/2/3/4/5). It is noteworthy that all CtNF-YB subfamily members are core members of the interaction network, suggesting that the CtNF-YA1 and CtNF-YB subfamilies may be central members in the regulation of safflower growth and development.

### 2.8. GO Enrichment Analysis of the CtNF-Y Gene Family

**Figure 6:**
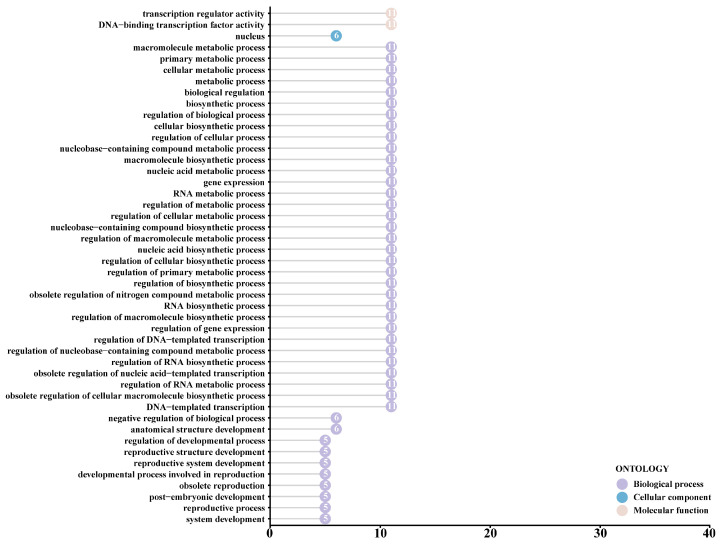
GO annotation analysis of the *CtNF-Y* gene family in safflower. GO—Gene Ontology.

Functional annotation of the *CtNF-Y* gene family was performed by a GO analysis, and the GO structure showed that the functions of the family genes were mainly enriched in 46 subclasses under three major categories, namely, biological processes, cellular organisation, and molecular functions (Figure 6). In the biological process category, all family members have 33 processes, including metabolic regulation and biosynthesis. In the classification of cellular components, the nuclear component is common to all members. In the molecular function classification, the molecular functions of the *CtNF-Y* gene family were mainly enriched in the functions of transporter and DNA-binding transcription factor. The results of GO enrichment indicated that *CtNF-Y* genes play an important role in the transport of substances in safflower.

### 2.9. Transcriptomics and qRT-PCR Analysis of CtNF-Y Genes

**Figure 7:**
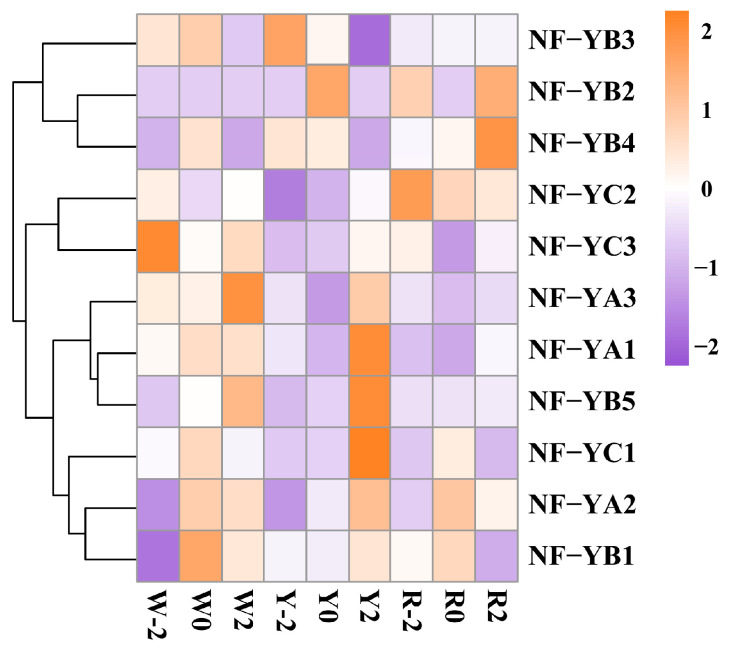
Transcriptome data analysis of *CtNF-Y* genes. Expression patterns of *CtNF-Y* gene family members were analysed in different flower colours (white—W, yellow—Y, and deep red—R) and at different times of bloom two days before (−2), on the day of bloom (0), and two days after bloom (2).

To investigate the relationship between *CtNF-Y* gene family members and safflower flower colour, based on the aforementioned protein-protein interaction (PPI) network prediction results, publicly available transcriptome data (Supplementary Figure S5) [32], and transcriptome data generated in our laboratory (Figure 7), we observed that CtNF-YA1, CtNF-YB1 and CtNF-YB4 are located within the protein interaction network, indicating that these three proteins are core members of the NF-Y family. In addition, these three genes exhibit significant expression differences in different flower colours and developmental stages. Therefore, these three genes were selected as the focus of subsequent experimental studies.

**Figure 8:**
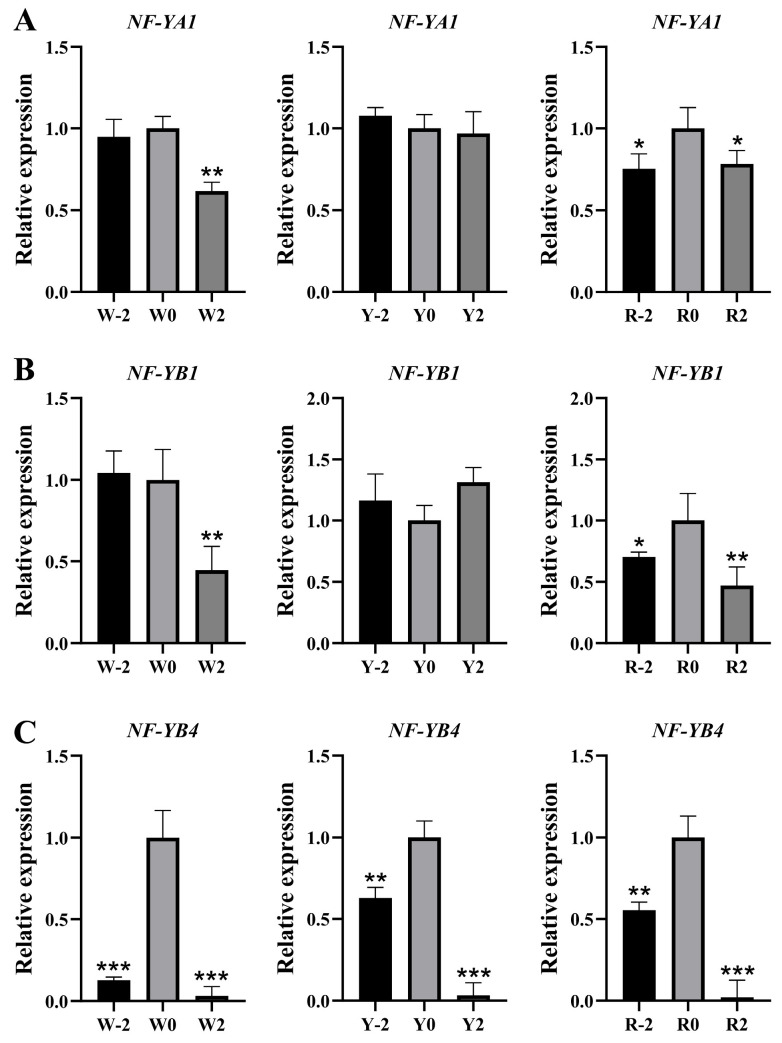
Expression of three *CtNF-Y* genes in different flowering periods. (**A**) Expression of *CtNF-YA1* gene at different flowering stages; (two days before (−2), on the day of bloom (0), and two days after bloom (2); white—W, yellow—Y, and deep red—R). (**B**) Expression of *CtNF-YB1* gene at different flowering stages. (**C**) Expression of *CtNF-YB4* gene at different flowering stages. Data were normalised to Ct60s. Vertical bars for qRT-PCR indicate the standard deviation, while an asterisk indicates the summary *p*-value of the independent samples *t*-test of the corresponding gene compared to the control (* *p* < 0.05, ** *p* < 0.01, *** *p* < 0.001).

To investigate the effect of the *CtNF-Y* gene family on flowering time, we analyzed the transcriptional levels of *CtNF-YA1*, *CtNF-YB1*, and *CtNF-YB4* in the same safflower variety at different time points. Compared with W0, Y0, and R0 (control group), we found that the expression level of the *CtNF-YA1* gene was lower in the W2 stage than in the W-2 and W0 stages, but higher in the R0 stage than in the other two stages. However, there were no significant differences between the Y-2, Y0, and Y2 stages. The expression trend of the *CtNF-YB1* gene was similar to that of *CtNF-YA1*, and the expression trends of *CtNF-YB1* and *CtNF-YA1* generally align with the transcription profile data (Figure 7). However, the expression trend of *CtNF-YB4* differs significantly from those of *CtNF-YA1* and *CtNF-YB1*, with expression levels in W0, Y0, and R0 being significantly higher than in the other two stages (Figure 8). Next, we analysed the expression patterns of these three genes in different flower colours during the same period. Compared with Y-2, Y0, and Y2 (control), the gene expression of *CtNF-YA1*, *CtNF-YB4* was found to be up-regulated in R-2, R0, and R2, relative to the expression of this gene in the other two flower colours. The results obtained from this study were consistent with the transcriptome data (Figure 7). The gene expression of *CtNF-YB1* in R2 was lower than that in Y2, but the gene expression of *CtNF-YB1* in R-2 and R0 was higher than that of *CtNF-YB1* in Y-2 and Y0 (Supplementary Figure S6). These results indicated that different varieties of safflower and different flowering periods have an impact on the expression patterns of these three *CtNF-Y* transcription factors.

### 2.10. Agronomic Traits and HSYA Content of Safflower

**Figure 9:**
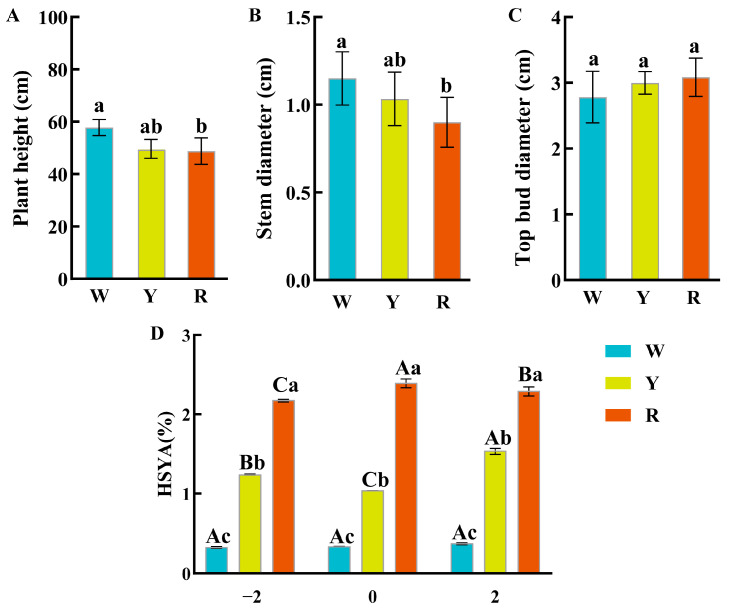
Agronomic traits and content of active components (HSYA) in different varieties of safflower under different flowering periods. (**A**) Plant height was measured in three safflower varieties (White—W, Yellow—Y, and Red—R) under flowering periods; (**B**) stem diameter; (**C**) terminal bud diameter; and (**D**) determination of Hydroxysafflor yellow A (HSYA) content in different safflower varieties at different flowering stages (two days before flowering (−2), on the day of flowering (0), two days after flowering (2)). Different colours represent different types of safflower. Different lowercase letters indicate differences between varieties with the same flowering period, while different uppercase letters indicate differences between varieties with different flowering periods.

We measured the plant height (Figure 9A), stem diameter (Figure 9B), and diameter of the terminal bud (Figure 9C) of three types of safflower at flowering stages. The results showed that there were significant differences in plant height, stem thickness, and terminal bud diameter among the different varieties of safflower. The white variety had significantly higher plant height and thicker stems than the red variety. There were no significant differences in terminal bud diameter among the three varieties. We then used HPLC to determine the content of Hydroxysafflor yellow A (HSYA) in the three types of safflower at different flowering stages. The results showed that HSYA content was higher in R than in Y and W at different flowering stages (Figure 9D).

### 2.11. Subcellular Localisation Analysis of CtNF-Y Proteins

**Figure 10:**
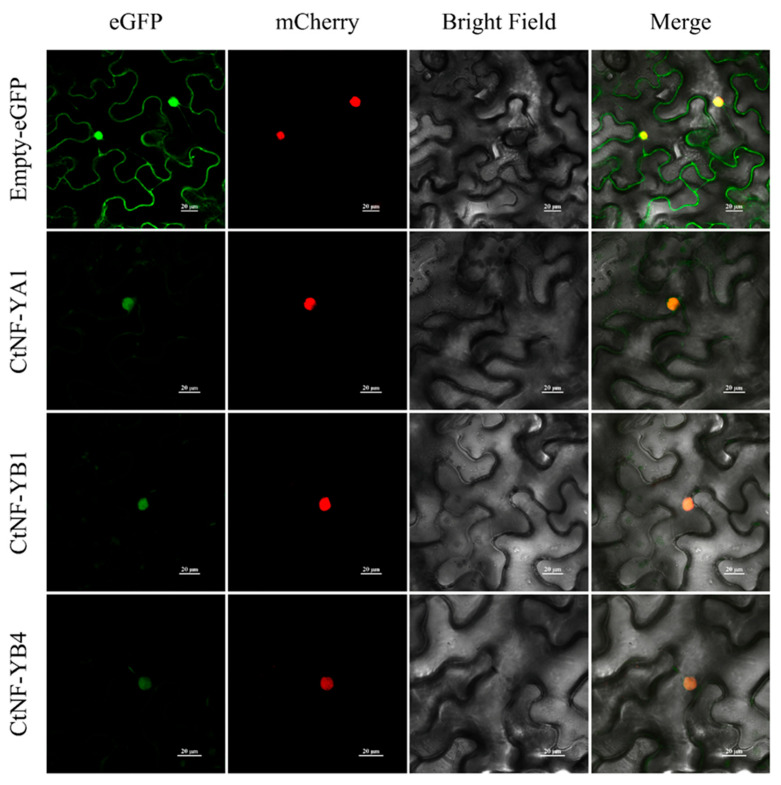
Subcellular localisation of CtNF-Y proteins in *Nicotiana tabacum* leaves. pCAMBIA1300-35S–eGFP and pCAMBIA1300-35–CtNF-Y–eGFP fusion proteins were transiently expressed in *N. tabacum* leaves and used confocal microscopy to determine their localisations. The nucleus was visualised with mCherry-labelled nuclear markers. Bar = 20 µm.

*Agrobacterium tumefaciens* containing CtNF-YA1-eGFP, CtNF-YB1-eGFP, and CtNF-YB4-eGFP were transferred into tobacco and transiently expressed. It was found that the green fluorescent signals of the above three proteins were concentrated in the nucleus of the cell (Figure 10), which was consistent with the results predicted on the Cell-PLoc website.

## 3. Discussion

Safflower is a multifunctional cash crop whose flowers are rich in pigments and flavonoids, which can be widely used in dyeing and weaving and medicine, and whose seeds are rich in oils, proteins, and vitamins, which can be used in food and health care industries, etc. In recent years, significant progress has also been made in elucidating the biological functions of NF-Y in plants. The *NF-YA3* and *NF-YA8* genes in *Arabidopsis* participate in the formation and development of early plant embryos, and mutations in these genes lead to embryonic defects [33]. The specific mechanism involves the binding of miRNA (miR169) and members of the NF-Y family (NF-YA1, 3, 5, 8, and 10) in *Arabidopsis*, which positively regulates plant cell embryogenesis (SE) [34]. In addition, NF-Y plays an important role in biological stress, abiotic stress, and plant hormone responses. Overexpression of transcription factor *NF-YC9* enhances *Arabidopsis* sensitivity to abscisic acid [35]. Overexpression of *NF-YB2* and *NF-YB3* specifically enhanced the drought tolerance and heat tolerance of *Arabidopsis*, respectively [21]. Studies have shown that AtHAP5A can bind to the CCAAT motif of AtXTH21 and regulate the frost resistance of *Arabidopsis* [22]. There are also some studies that have found the 35S: AtNFYA1 overexpression system exhibits hypersensitivity to salt stress and abscisic acid (ABA) during the early post-germination growth stage. Compared with the wild type (WT), the transgenic system exhibits severe post-germination growth arrest under salt stress and ABA treatment, indicating that *AtNFYA1* negatively regulates *Arabidopsis*’ response to salt stress [36]. Research has revealed that OsNF-YAs reduce rice resistance to *rice stripe virus* (RSV, *Tenuivirus*) and *southern rice black-streaked dwarf virus* (SRBSDV, *Fijivirus*) by inhibiting the MeJA signaling pathway [37]. ZmNFYA01 and ZmNFYA06 in maize (*Zea mays*) positively regulate maize resistance to *Setosphaeria turcica* and *Cochliobolus heterostrophus* [38]. Moreover, MeNF-YC15 can positively regulate the resistance of cassava (Manihot esculenta Crantz) to cassava bacterial blight (CBB) [39]. Although there are many reports on *NF-Y* transcription factors in other plants, there is still a lack of reports on safflower *NF-Y* transcription factors.

In this study, we identified 11 *CtNF-Ys* transcription factors using bioinformatics methods. Conservative motifs and domains are often associated with protein function [40]. In this study, we found that CtNF-YA, CtNF-YB, and CtNF-YC also share similar motifs, domains, and gene structures, indicating that members of this family have remained relatively conserved during evolution. Although numerous reports have described the functions of NF-Y transcription factors, the most widely recognised biological function of NF-Y is its role as a light-cycle-dependent positive regulator of flowering. Additionally, NF-Y is involved in plant photomorphogenesis [41,42,43]. In this study, we analyzed the cis-acting elements of the *CtNF-Y* transcription factor and found that, in the three subfamilies—*CtNF-YA*, *CtNF-YB*, and CtNF-YC—almost all contain light-responsive elements, such as the G-box, ACE, AT1-motif, and ATC-motif. Phylogenetic analyses showed that CtNF-YB1 and CtNF-YB4 were located in the same cluster as AtNF-YB2 and AtNF-YB3 in *A. thaliana*, as well as with PtNF-YB1 in poplar, and CtNF-YA1 was located in the same cluster as AtNF-YA1 in *Arabidopsis thaliana*, indicating that these genes are evolutionarily related and may have similar functions. Moreover, it has been found that AtNF-YB2 and AtNF-YB3 can control flowering time in *Arabidopsis* and have the function of promoting flowering under long sunlight [18]. Overexpression of *AtNF-YA1* can delay the flowering time of *A. thaliana* to a certain extent [44], and poplar PtNF-YB1 can make plants flower earlier [45]. Suggesting that CtNF-Y transcription factors may have a role in regulating flowering. In this study, we revealed a protein interaction network comprising 457 edges and 31 nodes. Among them, six members of the CtNF-Y family (CtNF-YA1, CtNF-YB1, CtNF-YB2, CtNF-YB3, CtNF-YB4, and CtNF-YB5) were identified as participating in this interaction network, suggesting that they may be core members of the CtNF-Y family involved in the regulation of safflower growth and development. Based on published transcriptomic data (Supplementary Figure S5) and transcriptomic data from our laboratory (Figure 7), we found that the expression levels of CtNF-YA1, CtNF-YB1, and CtNF-YB4 showed significant differences among safflower varieties with different flower colours and at different flowering stages. Based on this, this study selected these three genes as the focus of further experimental research.

Flower colour is one of the important traits of safflower. Previous studies have reported significant differences in the gene expression patterns of the *CtNF-Y* gene family among safflower varieties with different flower colours [32]. Our study found that the expression levels of the *CtNF-YA1* and *CtNF-YB4* genes in red-flowered varieties were significantly higher than those in white-flowered and yellow-flowered varieties. However, the expression pattern of the CtNF-YB1 gene was inconsistent with the trends of the other two genes. It was observed that two days before flowering and on the day of flowering, the expression levels of the *CtNF-YB1* gene in red-flowered varieties were higher than those in white-flowered and yellow-flowered varieties. However, two days after flowering, the expression levels of the *CtNF-YB1* gene in red-flowered varieties were lower than those in yellow-flowered varieties. Therefore, we speculate that the *CtNF-YA1*, *CtNF-YB1*, and *CtNF-YB4* genes may be associated with the formation of different flower colours. However, we learned from the literature that *NF-Y* transcription factors also regulate plant flowering time [20]. Therefore, we further investigated the effect of flowering time on the expression patterns of *CtNF-YA1* and *CtNF-YBs* (1 and 4). In red-flowered varieties, the expression levels of the *CtNF-YA1* and *CtNF-YB1* genes were significantly higher on the day of flowering, while the expression level of the *CtNF-YB4* gene was significantly higher on the day of flowering in all three flower colours (red, yellow, and white), showing a trend of first increasing and then decreasing. This trend is somewhat similar to the transcriptomic data (Figure 7), leading us to speculate that these genes may be involved in regulating the flowering time of red flowers in different varieties. Subcellular localisation analysis showed that the three transcription factors CtNF-YA1, CtNF-YB1, and CtNF-YB4 are localised to the cell nucleus, indicating that they function in the nucleus, consistent with the results predicted by Go analysis. This study successfully identified three candidate genes that may regulate red flower colour formation and flowering time based on published transcriptomic data and bioinformatics methods. Preliminary experiments validated the influence of flower colour and flowering time on the expression patterns of these three candidate genes, providing a theoretical basis for further investigations into the mechanisms regulating red flower colour and flowering time.

## 4. Materials and Methods

### 4.1. Plant Materials

Grow safflower in pots, three of each flower colour (red, yellow, and white). Wait for the safflower to grow to full bloom, collect filaments two days before (−2), on the day of bloom (0), and two days after bloom (2), and store at −80 °C for later use (Supplementary Figure S7).

### 4.2. Identification and Characterisation Analysis of the CtNF-Y Gene Family

Our laboratory obtained the complete genome and annotation information of safflower. The protein sequences of all CtNF-Y members from *Arabidopsis thaliana* were downloaded from the TAIR (https://www.arabidopsis.org/, accessed on 1 February 2025) [46], and those from *Helianthus annuus* were obtained from the NCBI database (https://www.ncbi.nlm.nih.gov/, accessed on 2 February 2025) [47]. Eleven CtNF-Y transcription factors were identified in safflower, and their sequences are listed in Supplementary Tables S3 and S4. These sequences were aligned (e-value 1 × 10^−5^) against the safflower genome in our laboratory to identify homologous sequences, which were retained as candidate CtNF-Y family protein sequences. The Pfam (http://Pfam.sanger.ac.uk/, accessed on 3 February 2025) [48] and SMART (https://smart.embl.de/smart/set_mode.cgi?GENOMIC=1, accessed on 4 February 2025) [49] online analysis tools were used to annotate the domains of the candidate sequences. Sequences containing NF-YA(PF02045), NF-YB, and NF-YC(PF00808) domains were ultimately identified as the candidate CtNF-Y sequences in safflower.

In addition, the basic physicochemical properties, such as amino acid sequence length, protein molecular weight (Mw), theoretical isoelectric point (pI), and hydrophilicity of CtNF-Ys, used the ExPASy website (https://web.expasy.org/compute_pi/, accessed 10 February 2025) [50] for analysis. Prediction of the signal peptides and transmembrane domains of CtNF-Y proteins used the Signal 6.0 (https://services.healthtech.dtu.dk/services/SignalP-6.0/, accessed 10 February 2025) [51] and TMHMM 2.0 websites (https://services.healthtech.dtu.dk/services/TMHMM-2.0/, accessed 11 February 2025) [52]. The subcellular localisation of CtNF-Y proteins was analyzed using the Cell-PLoc 2.0 tool (http://www.csbio.sjtu.edu.cn/bioinf/Cell-PLoc-2/, accessed 12 February 2025) [53]. The secondary and three-dimensional structures were predicted by the online websites SOPMA (https://npsa-prabi.ibcp.fr/cgi-bin/npsa_automat.pl?page=npsa_sopma.html, accessed 12 February 2025) [54] and SWISS-MODEL (https://swissmodel.expasy.org/interactive, accessed 12 February 2025) [55].

### 4.3. Phylogeny, Gene Structure, and Conserved Motif Analysis of CtNF-Ys

First, the multiple sequence alignment of full-length CtNF-Y amino acid sequences was performed using the ClustalW program in MEGA 11, and they were further analyzed in GeneDoc v2.7.000 software [56]. Then, the phylogenetic tree was constructed using the Neighbour-Joining (NJ) method with 1000 bootstrap replications in MEGA 11 [57]. Exon/intron sites and length information were extracted from the respective genome annotation GFF files of the safflower, and the structure drawings were scaled and displayed using the Gene Structure View (Advanced) in TBtools v1.099 (Toolbox for Biologists) [58]. The conserved protein motif of the safflower family was identified using MEME Suite v.5.5.5 (https://meme-suite.org/meme/tools/meme, accessed on 13 February 2025) [59], with a maximum number of 15 motifs.

### 4.4. Identification of Cis-Regulatory Elements in the Promoters of CtNF-Y Genes

Cis-acting elements were obtained from the 2000 bp upstream regions of the *CtNF-Y* genes. The prediction was made using the PlantCARE website (http://bioinformatics.psb.ugent.be/webtools/plantcare/html, accessed on 28 October 2023) [60]. The distribution map of cis-acting elements and the heatmap were generated using TBtools [61]. The detected elements were divided into different response types based on their annotated functions.

### 4.5. Chromosome Distribution and Collinearity Analysis of CtNF-Y Genes

Chromosomal localisation and collinearity analysis were performed with TBtools. The chromosomal localisation was performed by Gene Location Visualize from GTF/GFF, and collinearity analysis was conducted using the One Step MCScanX-Super Fast toolkit [62].

### 4.6. Protein Interaction Network and GO Enrichment Analysis of the CtNF-Ys

Interactions of CtNF-Y family protein members in safflower were analyzed using the STRING website (https://cn.string-db.org/, accessed on 15 December 2024) [63]. The protein sequence analysis confidence was set to 0.4, and the output results were visualised using Cytoscape (Version 3.10.0) [64]. GO enrichment analysis was performed using TBtools [65].

### 4.7. Transcriptome Data Analysis of CtNF-Y Genes

The transcriptomic data used in this study were analysed based on existing transcriptomic data (not yet publicly available) from our laboratory. For RNA-seq analysis, total RNA was isolated from safflower inflorescences using TRIzol reagent (Invitrogen, Oxford, UK), followed by the precise detection of RNA integrity and total RNA content using an Agilent 2100 bioanalyzer (Agilent Technologies, Santa Clara, CA, USA). cDNA library construction and sequencing were performed by Novogene. Specifically, mRNA was enriched from total RNA using Oligo dT magnetic beads. After fragmentation, the first strand of cDNA was synthesised using random hexameric primers, followed by synthesis of the second strand of cDNA. After end repair, A-tailing, adapter ligation, fragment selection, amplification, and purification, the library was ready for use. After construction, the library was initially quantified using a Qubit 2.0 Fluorometer, diluted to 1.5 ng/μL, and then the insert size of the library was detected using an Agilent 2100 Bioanalyzer. After confirming that the insert size met expectations, qRT-PCR was used to accurately quantify the effective concentration of the library (the effective concentration of the library was higher than 1.5 nM) to ensure library quality. After the library passes inspection, different libraries are pooled according to their effective concentration and the required downstream data volume and then sequenced using Illumina. HISAT2 v2.0.5 was used to build an index for the reference genome, and HISAT2 v2.0.5 was used to align paired-end clean reads with the safflower reference genome (The chromosome-scale reference genome of safflower provides insights into linoleic acid and flavonoid biosynthesis).

New gene prediction was performed using StringTie (1.3.3b) [66]. StringTie (ISAT: a fast spliced aligner with low memory requirements; StringTie enables improved reconstruction of a transcriptome from RNA-seq reads.) uses a network flow algorithm and optional de novo assembly to assemble transcripts. featureCounts (1.5.0-p3) is used to calculate the number of reads mapped to each gene. FPKM values were then calculated for each gene based on its length, and the number of reads mapped to that gene was calculated. Differential expression analysis between two conditions/groups was performed using the DESeq2 R package (1.20.0). A corrected *p*-value ≤ 0.05 and |log2(fold change)| ≥ 1 were set as the threshold for significant differential expression. Annotation of deg was performed using public databases for enrichment analysis (de novo sequencing and transcriptome analysis of the desert shrub, *Ammopiptanthus mongolicus*, during cold acclimation using Illumina/Solexa (San Diego, CA, USA)). Finally, we used TBtools v1.099 to analyse the expression patterns of 11 *CtNF-Y* gene family members at different flower colours and flowering stages, and generated heat maps. Specific transcriptomic data are provided in Supplementary Table S5.

### 4.8. qRT-PCR Analysis of CtNF-Y Genes

We performed Quantitative Real-time PCR (qRT-PCR) analysis using the Real Time PCR EasyTM-SYBR Green I Mix on the Roche LightCycler 480 (Basel, Switzerland) instrument. The reaction volume was 20 µL. RNA was provided by Novogene Company (Beijing, China). The reverse transcription cDNA kit used was HiScript IIQ RT SuperMix for qPCR (+gDNA wiper) (sourced from Nanjing Wazim Biotechnology Co., Ltd., Nanjing, China). After testing, the average concentration of cDNA required for this study was 85 ng/μL. All qRT-PCR primer sequences were designed with Primer 5.0 software (PREMIER Biosoft, San Francisco, CA, USA) and are shown in Supplementary Table S6.

To ensure the accurate normalisation of the gene expression data, we used a stable and reliable reference gene, the *Ct60s* gene (GenBank accession: KJ634810.1), as an internal control [67]. In addition, gene expression levels were quantified as relative fold change. Each reaction was conducted with three biological replicates. The obtained qRT-PCR data were analyzed using the 2^−∆∆CT^ method, a widely accepted approach for relative quantification of gene expression [68]. The bar graph was drawn by GraphPad Prism (version 9.0, GraphPad Software, San Diego, CA, USA).

### 4.9. Determination of the Content of Active Components in Safflower

The content of active components (HSYA) in safflower flowers was determined by high-performance liquid chromatography (HPLC) [69]. The chromatographic column used was an Agilent EC-C18 column (150 mm × 4.6 mm, 4 µm). The detection wavelength was set to 403 nm. The mobile phase consisted of A: water, B: methanol, C: acetonitrile, and D: 0.4% phosphoric acid solution. The elution conditions were as follows: 0–60 min, 5% B and 95% D; 60–65 min, 95% B and 5% D; and 65–70 min, 5% B and 95% D. The column temperature was maintained at 30 °C, and the injection volume was 10 µL. The standard concentrations were 0.424 mg/mL.

### 4.10. Subcellular Localisation Assay of CtNF-Y Proteins

The coding sequences (CDS) of the CtNF-Ys were amplified using primers that removed terminators but contained arms homologous to the expression vector, and the target fragment was recovered. Then, the sequences were cloned into the KpnI and SalI digested pCAMBIA1300–eGFP vector by homologous recombination. Three recombinant vectors included 35S–CtNF-YA1–eGFP, 35S–CtNF-YB1–eGFP, and 35S–CtNF-YB4–eGFP, along with the empty vector 35S–1300–eGFP. The gene cloning primer sequence is shown in Supplementary Table S7.

The constructed vector was transformed into *Agrobacterium rhizogenes* GV3101, infiltrated into four-week-old *Nicotiana benthamiana* leaves, and dark-incubated for 48 h. The fluorescence signals of the epidermis of *N. benthamiana* leaves were observed using a Nikon Eclipse Ti2 ultrahigh-resolution confocal fluorescence microscope (Nikon, Japan). For GFP fluorescence and red fluorescence analyses, an excitation laser line of 488/561 nm was used. The experiments used pCAMBIA1300-35S-mCherry-NLS (Puint, Xi’an, Shaanxi, China) as a marker for the nucleus.

## 5. Conclusions

This study is the first genome-wide analysis of *NF-Ys* in safflower. A total of 11 CtNF-Y were identified on six chromosomes of safflower, and the CtNF-Y protein members were analyzed in terms of conserved motifs, conserved structural domains, gene structure, secondary and three-dimensional structure and phylogeny. These findings revealed a high degree of conservation and evolutionary relationships among the *CtNF-Y* genes. Collinearity analysis indicated that tandem duplications were the main drivers of the expansion of the *CtNF-Ys* gene family. Through protein interaction network analysis, we identified the core members of the CtNF-Y family (CtNF-YA1, CtNF-YB1, CtNF-YB4) and investigated their expression patterns under different flower colours at the same time. In addition, it was found that different flowering periods under the same flower colour resulted in significant changes in the expression patterns of *CtNF-YA1*, *CtNF-YB1*, and *CtNF-YB4* in safflower, which may be related to the involvement of *NF-Y* family genes in the regulation of safflower flowering time. The results of this study will provide a reference for further research on the molecular mechanism of safflower *CtNF-Y* genes in regulating the formation of flower colour and flowering time and will be of value to the breeding of safflower varieties.

## Data Availability

All data generated or analyzed during this study are included in this article. Further inquiries can be directed to the corresponding authors.

## Supplementary Materials

The following supporting information can be downloaded at: https://www.mdpi.com/article/10.3390/plants14142111/s1, Figure S1: Hydrophilic/hydrophobic analysis of all CtNF-Y members; Figure S2: Transmembrane domain analysis of all CtNF-Y members; Figure S3: Signal peptide analysis of all CtNF-Y members; Figure S4: Prediction of the three-dimensional structure of CtNF-Y proteins in safflower; Figure S5: Transcriptome data analysis of *CtNF-Y * genes; Figure S6: Expression of three *CtNF-Y* genes in different flower colours; Figure S7: Changes in flower colour and filament colour during different flowering periods; Table S1: Basic information of NF-Y genes identified in *C. tinctorius* L; Table S2: Secondary structure predictions of CtNF-Ys; Table S3: Nucleic acid sequence of *CtNF-Y* family members; Table S4: Amino acid sequence of CtNF-Y family members; Table S5: Transcriptome data size and quality control; Table S6: qRT-PCR primer sequence; Table S7: Gene cloning primer.
