# Supplementary material for: Genome-Wide Identification of the CtNF-Y Gene Family and Expression Analysis of Different Flower Colours and Different Flowering Stages in Carthamus tinctorius L
Source: Plants (Basel). 2025 Jul 9;14(14):2111. doi: 10.3390/plants14142111 (PMC12299961; doi:10.3390/plants14142111)
Supplement: Supplementary file 1 [file plants-14-02111-s001.zip › plants-3678567- CONVERSION REVISED Supplementary Figure.pdf]

**Supplementary Table 1.** Basic information of NF-Y genes identified in *C. tinctorius L.*

| Protein | Gene ID | Amino Acid | Molecular Weight (kDa) | Theoretical pI | Instability Index | Aliphatic index | GRAVY | PredictedLocation |
|---|---|---|---|---|---|---|---|---|
| NF-YA1 | *CtAH06T0161100.1* | 213 | 23.87 | 7.85 | 75.84 | 53.15 | -1.050 | Nucleus |
| NF-YA2 | *CtAH06T0254200.1* | 294 | 31.44 | 8.47 | 57.65 | 52.41 | -0.826 | Nucleus |
| NF-YA3 | *CtAH11T0162900.1* | 354 | 38.93 | 8.63 | 71.03 | 57.34 | -0.768 | Nucleus |
| NF-YB1 | *CtAH03T0049100.1* | 180 | 18.73 | 5.28 | 41.63 | 55.89 | -0.651 | Nucleus |
| NF-YB2 | *CtAH05T0060600.1* | 130 | 14.59 | 5.77 | 33.85 | 66.08 | -0.723 | Nucleus |
| NF-YB3 | *CtAH08T0064400.1* | 165 | 17.77 | 6.42 | 37.40 | 57.45 | -0.733 | Nucleus |
| NF-YB4 | *CtAH08T0094700.2* | 174 | 19.24 | 7.02 | 49.29 | 52.24 | -0.983 | Nucleus |
| NF-YB5 | *CtAH10T0195000.1* | 149 | 16.20 | 8.50 | 43.75 | 61.68 | -0.809 | Nucleus |
| NF-YC1 | *CtAH05T0054900.1* | 261 | 29.18 | 5.33 | 74.67 | 68.47 | -0.583 | Nucleus |
| NF-YC2 | *CtAH05T0182800.1* | 267 | 30.21 | 5.14 | 74.50 | 68.73 | -0.720 | Nucleus |
| NF-YC3 | *CtAH08T0199800.1* | 262 | 29.24 | 5.05 | 75.90 | 65.95 | -0.620 | Nucleus |

**Supplementary Table 2.** Secondary structure predictions of CtNF-Ys.

| Protein | Gene ID | α-Helix(%) | β-Turn(%) | Extended Strand(%) | Random Coil (%) |
|---|---|---|---|---|---|
| NF-YA1 | *CtAH06T0161100.1* | 15.9 | 3.29 | 1.41 | 79.34 |
| NF-YA2 | *CtAH06T0254200.1* | 12.59 | 3.06 | 1.02 | 83.33 |
| NF-YA3 | *CtAH11T0162900.1* | 21.75 | 5.37 | 1.98 | 70.90 |
| NF-YB1 | *CtAH03T0049100.1* | 37.78 | 3.33 | 3.89 | 55.00 |
| NF-YB2 | *CtAH05T0060600.1* | 56.15 | 0.77 | 3.85 | 39.23 |
| NF-YB3 | *CtAH08T0064400.1* | 42.42 | 1.21 | 3.03 | 53.33 |
| NF-YB4 | *CtAH08T0094700.2* | 39.66 | 0.00 | 4.02 | 56.32 |
| NF-YB5 | *CtAH10T0195000.1* | 45.64 | 0.00 | 4.70 | 49.66 |
| NF-YC1 | *CtAH05T0054900.1* | 31.42 | 1.53 | 1.15 | 65.90 |
| NF-YC2 | *CtAH05T0182800.1* | 31.46 | 5.62 | 1.87 | 61.05 |
| NF-YC3 | *CtAH08T0199800.1* | 30.92 | 1.53 | 1.15 | 66.41 |

**Supplementary Figure 1.** Hydrophilic/hydrophobic analysis of all CtNF-Y members.

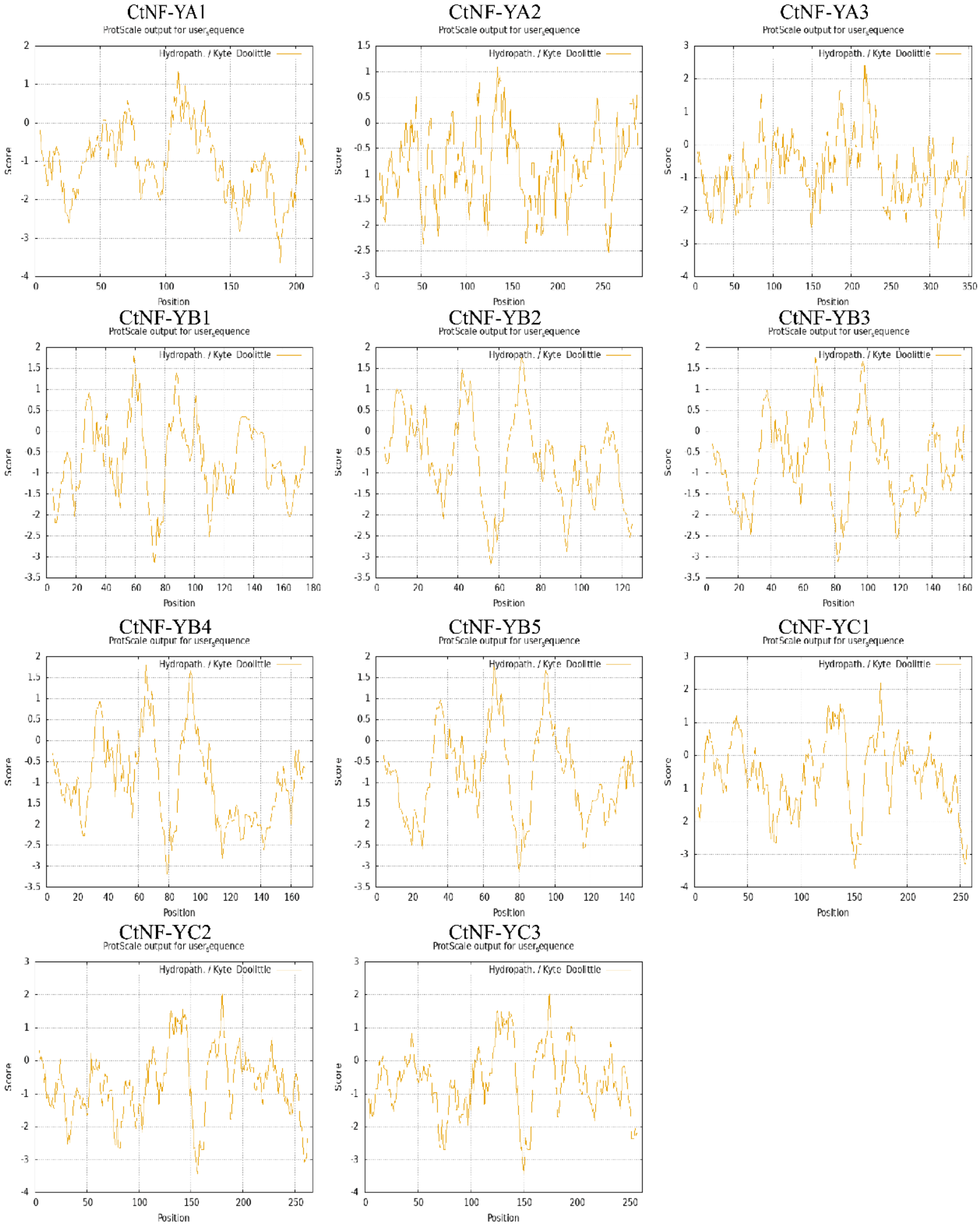


**Supplementary Figure 1.** Hydrophilic/hydrophobic analysis of all CtNF-Y members. The horizontal coordinate indicates each amino acid sequence, and the vertical coordinate indicates the hydrophilic index of the amino acid. The score less than 0 indicates that the amino acid is hydrophilic, otherwise the more positive the score, the more hydrophobic the amino acid located in that region of the protein.

**Supplementary Figure 2.** Transmembrane domain analysis of all CtNF-Y members.

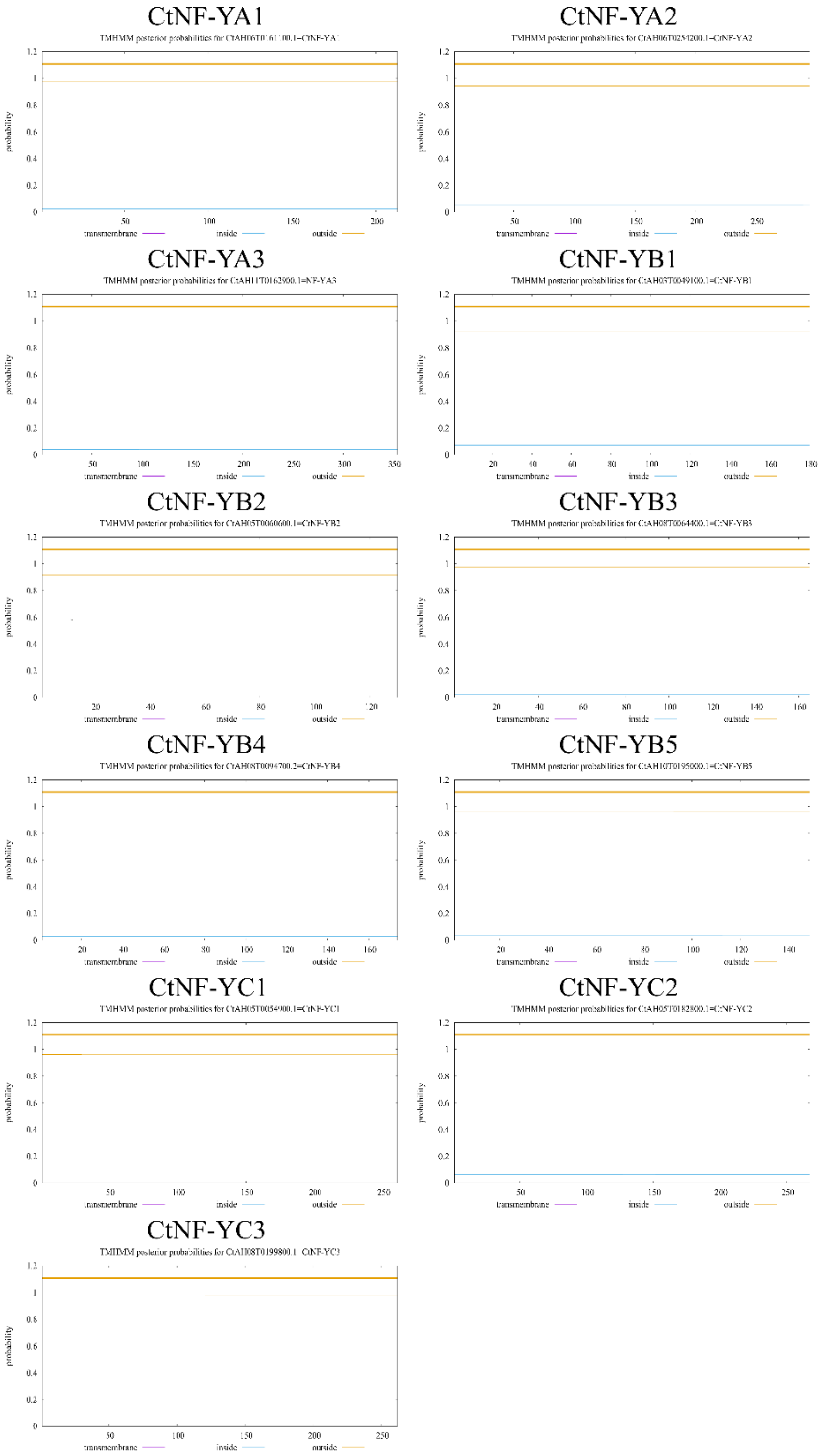


**Supplementary Figure 2.** Transmembrane domain analysis of all CtNF-Y members. The horizontal coordinate represents the amino acid sequence, and the vertical coordinate represents the probability of the region in which the protein sequence is located. The purple line represents the transmembrane region, the blue line represents the inside of the membrane, and the orange line represents the outside of the membrane.

**Supplementary Figure 3.** Signal peptide analysis of all CtNF-Y members.

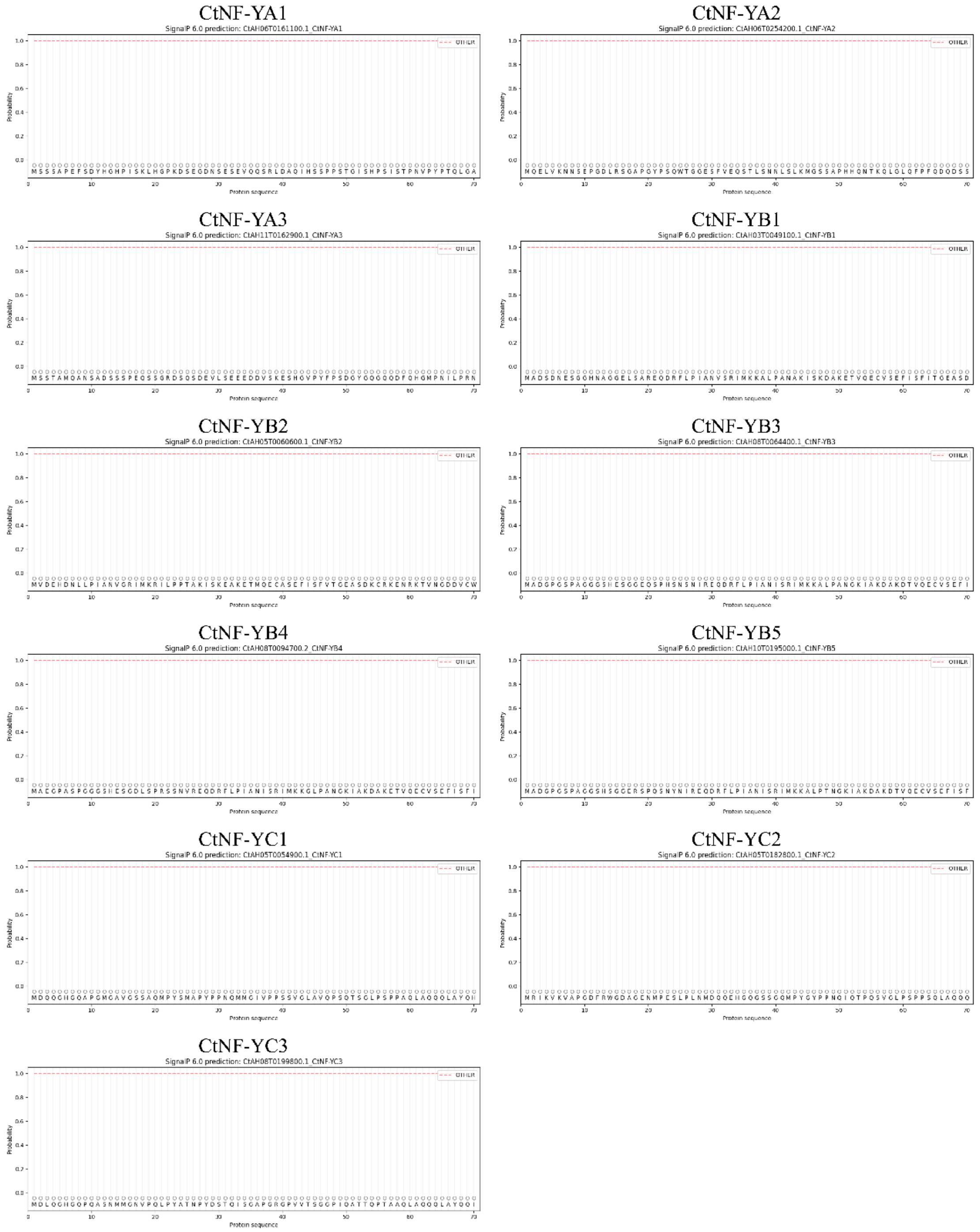


**Supplementary Figure 3.** Signal peptide analysis of all CtNF-Y members. The horizontal coordinate indicates the protein sequence, and the vertical coordinate indicates the presence or absence of a signal peptide.

**Supplementary Figure 4.** Prediction of the three-dimensional structure of CtNF-Y proteins in safflower.

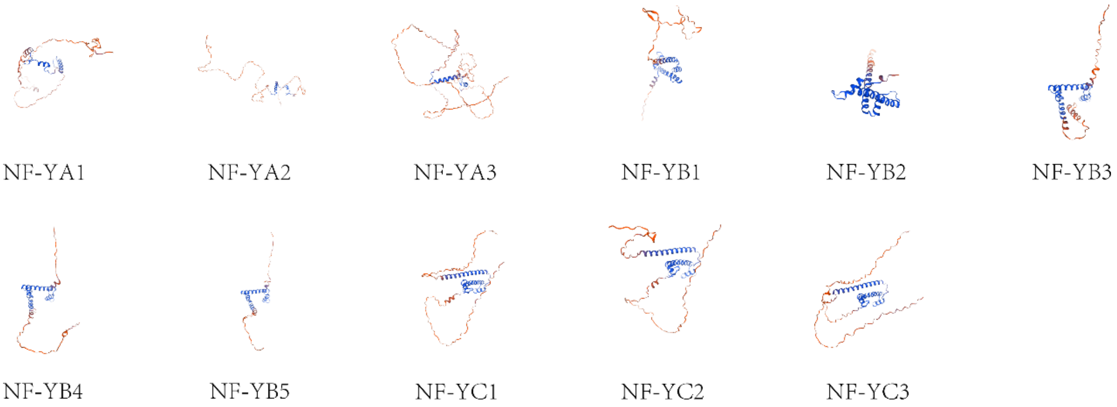


**Supplementary Figure 4.** Prediction of the three-dimensional structure of CtNF-Y proteins in safflower. The blue chain represents consistency with the template sequence and a higher score, while the red chain represents a lower score.

**Supplementary Figure 5.** Transcriptome data analysis of CtNF-Y genes.

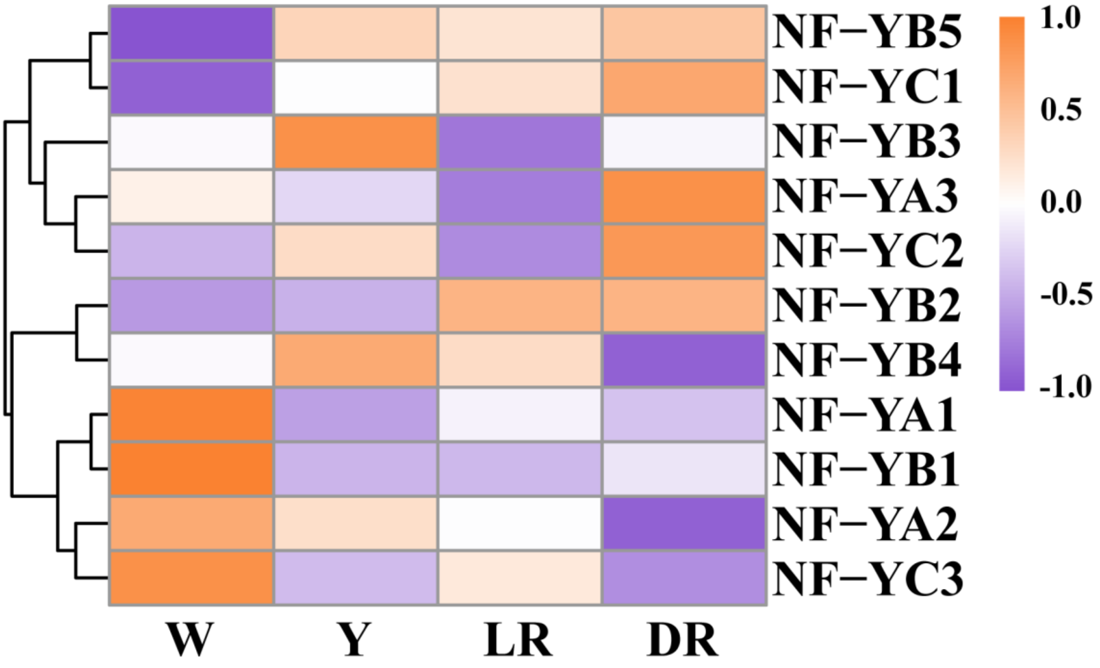


**Supplementary Figure 5.** Transcriptome data analysis of CtNF-Y genes. Expression patterns of CtNF-Y gene family members were analysed in different flower colours (white-W, yellow-Y , Light red-LR , and Deep red-DR).

**Supplementary Figure 6.** Expression of three *CtNF-Y* genes in different flower colours.

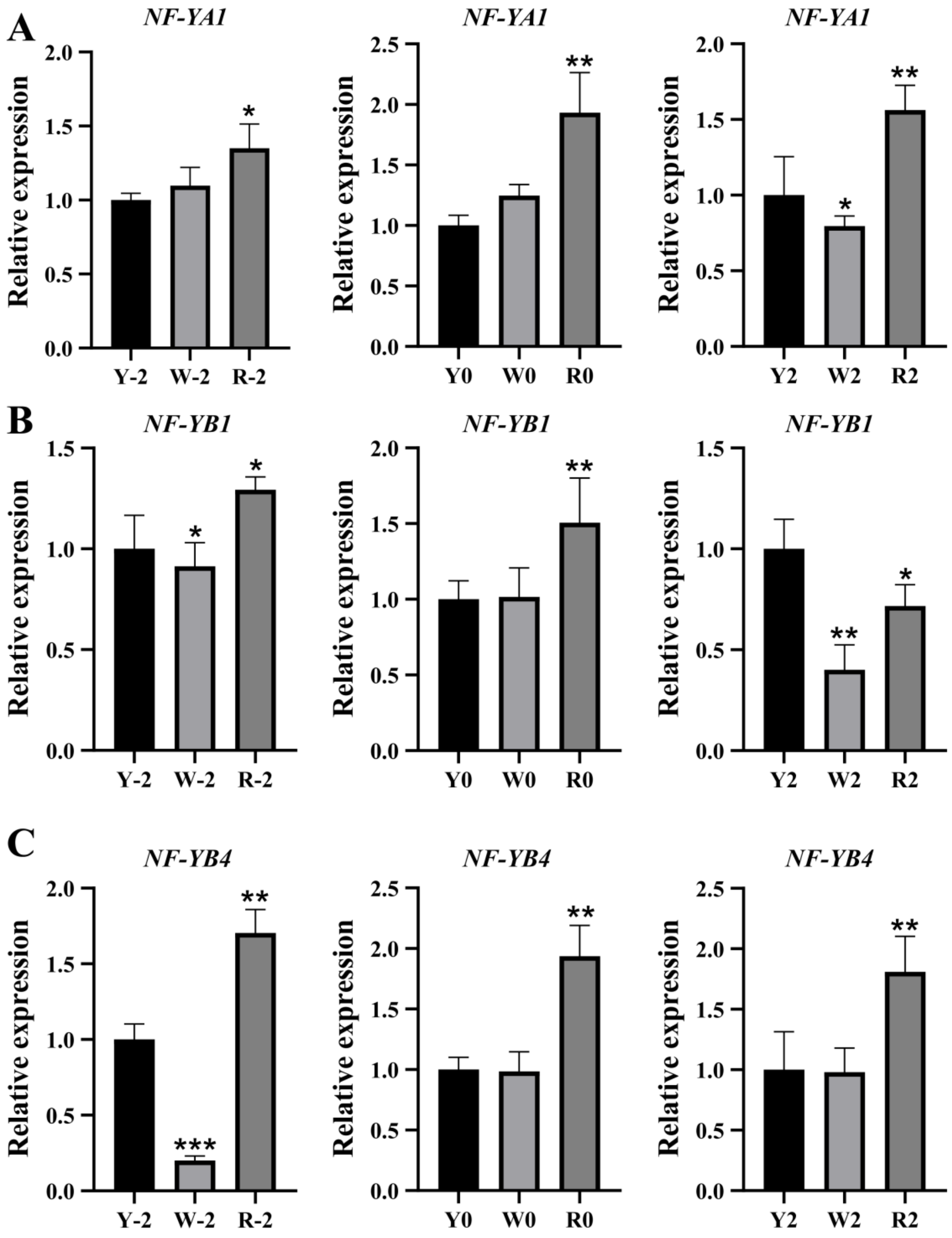


**Supplementary Figure 7.** Expression of three *CtNF-Y* genes in different flower colours. (A) Expression of *CtNF-YA1* gene in different flower colours (two days before (-2), on the day of bloom (0), and two days after bloom (2), white-W, yellow-Y, and deep red-R); (B) Expression of *CtNF-YB1* gene in different flower colours; (C) Expression of *CtNF-YB4* gene in different flower colors. Data were normalized to Ct60s. Vertical bars for qRT-PCR indicate the standard deviation, while an asterisk indicates the summary p-value of the independent samples t-test of the corresponding gene compared to the control (* $p < 0.05$, ** $p < 0.01$, *** $p < 0.001$).

**Supplementary Figure 7.** Changes in flower colour and filament colour during different flowering periods.

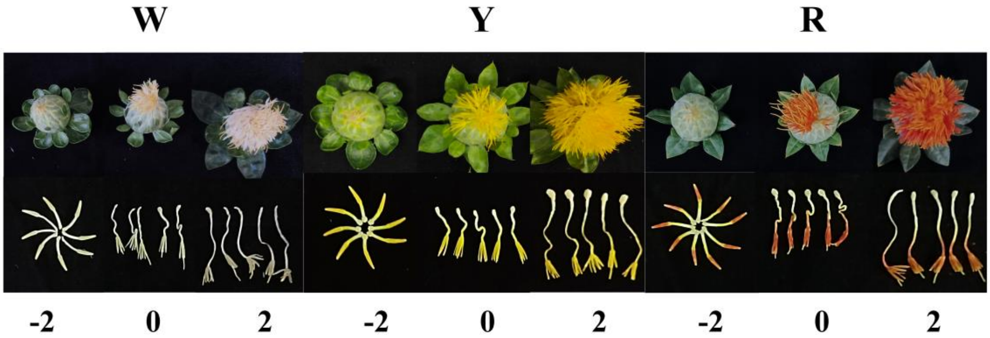


**Supplementary Figure 6.** Changes in red flower buds of different varieties under different flowering periods. R: red variety, W: white variety, Y: yellow variety. -2: two days before flowering, 0: day of flowering, 2: two days after flowering.
